# Understanding fluid administration approaches in children with co-morbidities and septic shock

**DOI:** 10.1186/s13054-017-1741-y

**Published:** 2017-08-02

**Authors:** Niranjan Kissoon

**Affiliations:** 1 Department of Pediatrics, BC Children’s Hospital, University of British Columbia, B245, 4480 Oak Street, Vancouver, BC V6H 3V4 Canada; 2 Child & Family Research Institute (CFRI), Vancouver, British Columbia Canada; 3 Division of Critical Care, BC Children’s Hospital, University of British Columbia, Vancouver, British Columbia Canada

**Keywords:** Severe malnutrition, Children, Hypovolemic shock, Septic shock, Mortality, Myocardial function, Echocardiography, Fluid resuscitation, Low and middle income countries

Fluid infusions are given to children to treat cardiovascular compromise from shock in locations across the globe, where resources vary widely. The most common cause of cardiovascular compromise is septic shock. In low and middle income countries severe malnutrition complicates sepsis largely from malaria, pneumonia, and diarrheal diseases, leading to interesting treatment conundrums. The high mortality rate of about 40% in children with severe malnutrition and shock from diarrheal diseases results from the confluence of clinical factors, inequity, socio-economic and cultural context resulting in late presentation, increased vulnerability, and sub-optimal care [1]. Fluid resuscitation in this context is risky with little margin for error, where small deficits or excesses can lead to cardiovascular collapse or respiratory failure. Thus, the WHO recommends a cautious approach with frequent clinical monitoring. This recommendation is supported by weak evidence and accompanied by a suggestion that research is needed to determine optimal fluid management in children with severe malnutrition and shock [2].

In response to these uncertainties, Obonyo and colleagues investigated the cardiovascular response to two fluid infusion regimes (bolus and continuous infusion over 5 h) in severely malnourished children with hypovolemic shock due to diarrheal diseases with dehydration [3]. The authors concluded that the concern of compromised cardiac function and vulnerability to fluid overload in children with severe malnutrition and diarrheal diseases with dehydration is not supported by their findings. However, there was a high mortality rate at 48 h (36–44%) and 28 days (56–82%) [3]. The rationale for this study is partly based on the FEAST trial in which bolus fluid in febrile children in Africa resulted in worse outcomes [4]. However, the FEAST trial excluded children with dehydration from diarrheal diseases, few enrolled had septic shock, and almost 60% of the children suffered from malaria. The findings of the FEAST trial signals a cautious approach to fluid boluses in low resource settings, but the final chapter on fluid approaches in children with septic shock in any setting is not yet written.

Septic shock is a complex pathophysiological derangement; its expression depends on microbial agent virulence, systemic inflammation, endothelial and microcirculation disruption, primary and secondary immune derangements, and coagulation and parenchymal tissue insults [5, 6]. Endothelial barrier dysfunction occurs early in septic shock and whether it is amplified in SM is unclear [7]. Mortality in children with diarrheal disease and septic shock is higher (67%) than in controls with dehydration (14%). Those with septic shock are also more likely to receive a blood transfusion and mechanical ventilation, therapies which may not be available in low and middle income countries [8]. Thus, the distinction between hypovolemic and septic shock has tremendous implications regarding treatment and outcomes because those in septic shock are more likely to need mechanical ventilation which may not be available [9].

Poor outcomes in children with septic shock in resource-poor settings are likely due to multi-organ dysfunction of late sepsis and insults to the myocardium where intensive care support is limited. Robust cardiac contractility relies on the myocardial milieu where substrates such as magnesium, calcium, acid base balance, hemoglobin, temperature, and energy stores are normal. Levels of these substrates are commonly abnormal in critically ill children. Thus, the inability to monitor or treat electrolyte disturbances and acid base may result in higher mortality rates due to cardiovascular collapse [3, 10]. Where intensive care support is available, mortality has decreased such that experts have proposed a composite outcome measure which includes both mortality and long-term morbidity.

The optimal approach to fluid administration in both high and low income countries is not yet settled, with no rigorous study conducted in high income countries. The FEAST trial, the largest and most rigorous attempt to address approaches to fluid administration was done in low and middle income settings in Africa. Validated approaches to administration of fluid in septic shock are sorely needed in view of the myriad etiologies and pathophysiologic perturbations and the recent body of evidence pointing to positive fluid balance as a predictor of poor outcomes in critically ill children even when intensive care is available.

Fluid resuscitation in sepsis has endured a long history of success, starting with initial experiences with cholera. What is still unclear is how much fluid should be infused and how fast to replenish intravascular volume deficits in children with shock and complex co-morbidities. We need to challenge our present dogma regarding approaches to fluid resuscitation. For instance, we need to further explore the role of increased lactate in septic shock because direct tissue oximetry has failed to show hypoxia and tissue partial pressure of oxygen may be elevated [11]. Oliguria, which is widely used as a guide to fluid resuscitation on the presumption of renal hypoperfusion, has been challenged [12]. Understanding the potency of fluids to increase vascular volume and remain in the vascular compartment [13] may possibly lead to revision of the Starling equation. Whether more modest fluid volumes and earlier introduction of inotropes will decrease overload and improve outcomes are also being explored [14].

While impractical in most low and middle income settings, an in-depth understanding of the pathophysiology and response to fluid in shock will be best obtained using multimodal monitoring including clinical examination and invasive central venous pressure and echocardiographic monitoring [15]. Ideally, organ function and microvascular tissue perfusion and oxygenation should also be monitored to understand the evolution of multi-organ dysfunction, such as in malaria where both micro- and macrovascular dysfunction may be contributory factors.

Scientific evidence thrives by unearthing facts and burying opinions. Thus, we need randomized clinical trials if we are to further our understanding of fluid therapies. Each child with sepsis is unique and factors such as their genetic predisposition, the social economic context, the inciting agent, and the trajectory of the septic episode must be considered in enrolment to understand and transition from generic to personalized and ultimately precision therapies. Challenging the status quo is never easy, but it may be time to rewrite history.

## Abbreviations

FEAST Trial: Fluid Expansion As Supportive Therapy Trial
WHO: World Health Organisation
